# Intestinal Antibody Responses to 2 Novel Live Attenuated Type 2 Oral Poliovirus Vaccines in Healthy Adults in Belgium

**DOI:** 10.1093/infdis/jiaa783

**Published:** 2020-12-24

**Authors:** Elizabeth B Brickley, Ruth I Connor, Wendy Wieland-Alter, Joshua A Weiner, Margaret E Ackerman, Minetaro Arita, Chris Gast, Ilse De Coster, Pierre Van Damme, Ananda S Bandyopadhyay, Peter F Wright

**Affiliations:** Department of Infectious Disease Epidemiology, London School of Hygiene & Tropical Medicine, London, United Kingdom; Department of Pediatrics, Dartmouth-Hitchcock Medical Center, Lebanon, New Hampshire, USA; Department of Pediatrics, Dartmouth-Hitchcock Medical Center, Lebanon, New Hampshire, USA; Thayer School of Engineering, Dartmouth College, Hanover, New Hampshire, USA; Thayer School of Engineering, Dartmouth College, Hanover, New Hampshire, USA; Department of Virology II, National Institute of Infectious Diseases, Tokyo, Japan; PATH, Seattle, Washington, USA; Centre for the Evaluation of Vaccination, Vaccine and Infectious Disease Institute, University of Antwerp, Antwerp, Belgium; Centre for the Evaluation of Vaccination, Vaccine and Infectious Disease Institute, University of Antwerp, Antwerp, Belgium; Bill & Melinda Gates Foundation, Seattle, Washington, USA; Department of Pediatrics, Dartmouth-Hitchcock Medical Center, Lebanon, New Hampshire, USA

**Keywords:** poliovirus, live attenuated vaccine, mucosal immunity, intestinal antibodies, eradication

## Abstract

In a blinded phase 1 trial (EudraCT 2017-0000908-21; NCT03430349) in Belgium, healthy adults (aged 18–50 years) previously immunized exclusively with inactivated poliovirus vaccine were administered a single dose of 1 of 2 novel type 2 oral poliovirus vaccines (nOPV2-c1: S2/cre5/S15domV/rec1/hifi3 (n = 15); nOPV2-c2: S2/S15domV/CpG40 (n = 15)) and isolated for 28 days in a purpose-built containment facility. Using stool samples collected near days 0, 14, 21, and 28, we evaluated intestinal neutralization and immunoglobulin A responses to the nOPV2s and found that nOPV2-c1 and nOPV2-c2 induced detectable poliovirus type 2–specific intestinal neutralizing responses in 40.0% and 46.7% of participants, respectively.

## Teaser

In a phase 1 trial, 2 novel type 2 oral poliovirus vaccine candidates induced detectable poliovirus type 2–specific intestinal neutralizing responses in 40.0% and 46.7% of participants, respectively, suggesting potential utility for reducing circulating vaccine-derived poliovirus transmission under outbreak scenarios.

The world is approaching the eradication of polio. The certification on 25 August 2020 that the World Health Organization African Region has achieved wild-type poliovirus-free status marks an important milestone in the endgame. However, Afghanistan and Pakistan continue to experience local transmission with newly detected cases of wild-type poliovirus type 1. Of equal concern, >20 countries have reported cases with circulating vaccine-derived polioviruses (cVDPVs) in 2020.

Type 2 cVDPVs present a notable challenge to eradication efforts. In April and May 2016, the routine use of the type 2 component of the Sabin oral poliovirus vaccine (OPV2) was stopped globally, and the inactivated Salk poliovirus vaccine (IPV) became the primary public health tool for inducing immunity to poliovirus type 2. The aims of removing OPV2 from routine vaccination were to limit new emergences of type 2 cVDPVs, minimize risks of vaccine-associated paralytic poliomyelitis, and enhance the immunogenicity of the bivalent OPV (bOPV) against poliovirus types 1 and 3 with which OPV2 interfered. Both Sabin and Salk vaccines are capable of inducing robust serum immune responses that can limit viremia, inhibit entry into the central nervous system, and protect vaccinated individuals from paralytic polio. However, primary vaccination schedules based on inactivated vaccine, in contrast to live attenuated vaccine, fail to induce intestinal mucosal immune responses that are critical for inhibiting enteric poliovirus replication and preventing fecal-oral transmission [1–6].

The cessation of the routine use of OPV2 has therefore led to a population level decrease in intestinal mucosal immunity to type 2 polioviruses, which may facilitate the continued circulation of type 2 VDPVs, especially in regions with suboptimal IPV coverage. In outbreak scenarios where communities are experiencing rapid spread of type 2 cVDPVs and risks of paralysis, reintroduction of Sabin monovalent OPV2 (mOPV2) has thus been the only recourse for halting transmission. However, even in highly regulated short-term campaigns, mOPV2 has the potential to genetically mutate and seed further cVDPV outbreaks, perpetuating a vicious cycle that threatens eradication.

To counter the limitations of the Sabin OPV2, recent efforts have focused on developing novel OPV2 (nOPV2) candidates that are engineered to be more genetically stable and less likely to revert to a neurovirulent form. A 2017 blinded phase 1 trial in Belgium of healthy adults provided encouraging evidence of the safety and systemic immunogenicity of 2 nOPV2 candidates (nOPV2-c1: S2/cre5/S15domV/rec1/hifi3; nOPV2-c2: S2/S15domV/CpG40) [7]. Although both candidates induced serum neutralizing antibodies, the median duration and magnitude of nOPV2 viral shedding were approximately 2 times higher for nOPV2-c1 than for nOPV2-c2. Using stool samples collected serially from the 15 participants per candidate (30 in total) in this trial, the current study aimed to investigate the intestinal mucosal immune responses observed after oral administration of these nOPV2 candidates.

## METHODS

### Study Design and Participants

The study design, candidate vaccines, and outcomes of the parent clinical trial have been reported in detail [7]. Briefly, healthy volunteers, aged 18–50 years who were previously immunized exclusively with IPV (up to 6 doses), were recruited between 22 May and 22 August 2017 to participate in a blinded phase 1 clinical trial (EudraCT 2017-0000908-21; NCT03430349) of 2 novel live attenuated OPV2s with a 28-day isolated follow-up in a purpose-built containment facility, referred to as Poliopolis [8], at the University of Antwerp in Antwerp, Belgium. The vaccine candidates are monovalent live-attenuated type 2 polioviruses derived from a modified Sabin-strain type 2 infectious complementary DNA clone and propagated in Vero cells; both candidates were engineered to improve the genetic stability of their attenuation (for further details, see [7]).

After a medical and psychological screening, eligible participants were sequentially allocated into 2 groups based on the sealed envelope selection of the first participant, with the first 15 participants receiving nOPV2-c2 and the second 15 receiving nOPV2-c1. Participants were closely monitored for solicited and unsolicited adverse events. Blood samples were collected on days 0 and 28. Nasopharyngeal swab samples were collected on days 0, 3, 7, and the final day of containment. Stool samples were collected daily as possible.

### Laboratory Procedures

As part of the parent clinical trial, investigators at the US Centers for Disease Control and Prevention evaluated poliovirus type 2–specific neutralizing antibody responses in serum and nOPV2 virus shedding in nasopharyngeal swab and stool samples [7]. Titers of neutralizing antibodies were measured in serum samples using standardized procedures [9]. Poliovirus type 2 RNA was detected in nasopharyngeal swab and stool suspensions using a Sabin multiplex real-time reverse-transcription polymerase chain reaction assay [10]. The magnitude of infectious virus in reverse-transcription polymerase chain reaction–positive samples was measured as the Cell culture infectious dose 50% per gram of stool, using a modified World Health Organization cell sensitivity assay, as described elsewhere [9].

For the current study, investigators at Dartmouth-Hitchcock Medical Center in Lebanon, New Hampshire, measured poliovirus serotype–specific intestinal neutralizing activity and immunoglobulin A (IgA) binding in the same stool samples, using methods as described elsewhere [1]. Briefly, stool neutralizing activity was measured by limiting dilution inhibition of luciferase-expressing wild-type-derived polio pseudoviruses [11] and expressed as the titer needed to achieve 60% neutralization (titers >2 were considered detectable). Total and poliovirus type–specific IgA in stool were quantified using a multiplex microsphere assay developed by coupling monovalent IPVs to fluorescently coded magnetic microspheres.

### Ethical Considerations

At enrollment, all participants provided written informed consent, which included provisions for the use of samples in future polio-related studies. Ethical approval was provided by the hospital and university institutional review boards of Dartmouth-Hitchcock Medical Center, the University of Antwerp, and the Antwerp University Hospital.

## RESULTS

Poliovirus type–specific intestinal antibody responses were evaluated in 30 adult participants (83% male; mean age [standard deviation], 32.3 [9.4] years), using 94 stool samples from the 15 nOPV2-c1 recipients and 95 from the 15 nOPV2-c2 recipients. IgA was detected in stool from all participants, and the median concentration of total IgA in the first prevaccination stool was 9440 ng/mL (interquartile range, 1620–22 500 ng/mL). Whereas all participants had documented poliovirus type 2–specific serum neutralization on day 0 (median titer [interquartile range], 56.9 [36.0–181] for nOPV-c1 and 36.0 [22.6–90.5] for nOPV-c2), only 1 participant had detectable poliovirus type 2–specific stool neutralization (ie, a titer of 4) at baseline (Table 1).

**Table 1.** Poliovirus Type–Specific Serum and Intestinal Antibody Responses to the Novel Type 2 Oral Poliovirus Vaccine Candidates

		N (%) or Median (IQR)^a^		
Immune Marker by Sample Type	Day	nOPV2-c1 (n = 15)	nOPV2-c2 (n = 15)	*P* Value^b^
Serum samples: PV type 2 serum neutralization titer	0	56.9 (36.0–181)	36.0 (22.6–90.5)	.10
	28	1150 (576–1450)	724 (362–1150)	.27
Stool samples^c^				
Detectable PV type 2 stool neutralization, No. (%)^d^	0	1 (6.7)	0 (0)	.31
	14	4 (26.7)	4 (26.7)	>.99
	21	3 (20.0)	2 (13.3)	.62
	28	5 (33.3)	5 (33.3)	>.99
	All	6 (40.0)	7 (46.7)	.71
PV type 2 stool neutralization titer	0	2 (2–2)	2 (2–2)	.32
	14	2 (2–2.7)	2 (2–2.5)	.96
	21	2 (2–2)	2 (2–2)	.59
	28	2 (2–4)	2 (2–7)	.79
PV type 2 stool IgA MFI	0	11.3 (2.0–14.1)	12.0 (3.5–17.0)	.26
	14	6.8 (3.2–22.3)	7.4 (5.6–38.1)	.42
	21	10.8 (5.0–19.5)	10.0 (2.5–46.5)	.72
	28	14.5 (2.0–29.5)	12.3 (1.5–112)	.76
PV type 1 stool IgA MFI	0	6.3 (2.0–21.5)	13.0 (5.9–22.5)	.25
	14	8.0 (1.5–11.3)	9.0 (4.4–24.0)	.42
	21	4.3 (0–8.0)	3.5 (0–16.0)	.72
	28	6.3 (0–12.0)	4.5 (0–28.0)	.68
PV type 3 stool IgA MFI	0	5.9 (3.5–31.0)	13.8 (2.0–40.5)	.56
	14	3.5 (0–15.0)	8.3 (3.4–20.0)	.16
	21	0 (0–4.8)	6.0 (0–18.5)	.15
	28	2.0 (0–14.5)	1.0 (0–18.8)	.73

Abbreviations: IgA, immunoglobulin A; IQR, interquartile range; MFI, mean fluorescence intensity; nOPV2-c1and nOPV2-c2, novel type 2 oral poliovirus vaccine candidates; PV, poliovirus.

^a^Data represent median (IQR) values unless otherwise specified.

^b^ *P* values based on χ ^2^ or Mann-Whitney *U* tests.

^c^Because stool samples were not available for all participants on all days, we described the aggregate of the measurements obtained from samples collected days on − 2 to 0 (before vaccination) for day 0, days 12–16 for day 14, days 19–23 for day 21, and days 26–30 for day 28. If ≥2 stool samples were collected within each window for a given participant, we calculated the geometric mean of the stool neutralization titer or IgA MFI.

^d^We considered any poliovirus type 2–specific stool neutralization titer >2 to be detectable.

After vaccination, nOPV2 RNA was detected in stool from 100% (15 of 15) of participants who received nOPV2-c1 and from 87% (13 of 15) of participants who received nOPV2-c2, as reported elsewhere [7]. In contrast, poliovirus type 2–specific intestinal neutralizing responses in stool were detected (ie, with a titer >2 at ≥1 time point) after vaccination in only 40% (6 of 15) of nOPV2-c1 recipients and 47% (7 of 15) of nOPV2-c2 recipients (Table 1 and Supplementary Figure 1). No type 2–specific intestinal neutralizing responses were detected in the 2 participants with no detectable nOPV2 shedding. Baseline serum neutralizing titers did not appear to be correlated with subsequent stool neutralizing responses to the vaccines (Supplementary Figure 2).

**Figure 1.**
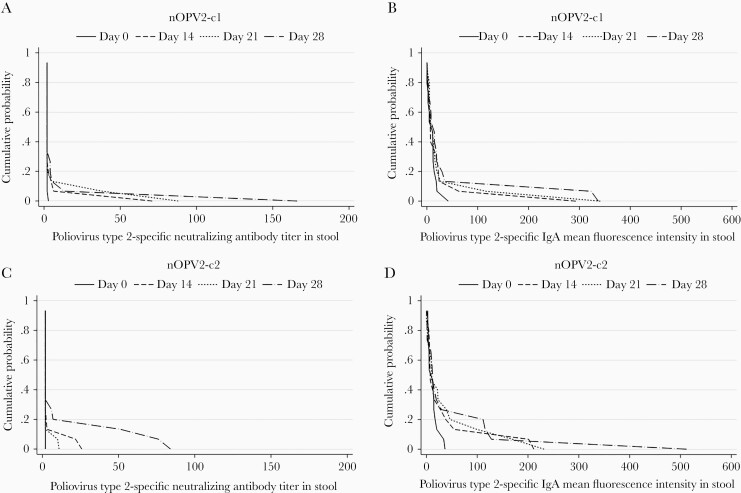
Reverse cumulative distribution functions of poliovirus type 2–specific stool neutralizing activity and immunoglobulin A (IgA) levels before and after receiving nOPV2-c1 (n = 15) (*A, B*) or nOPV2-c2 (n = 15) (*C, D*). Because stool samples were not available for all participants on all days, we described the aggregate of the measurements obtained from samples collected on days −2 to 0 (before vaccination) for day 0, days 12–16 for day 14, days 19–23 for day 21, and days 26–30 for day 28. If ≥2 stool samples were collected within each window for a given participant, we calculated the geometric mean of the stool neutralization titer or IgA mean fluorescence intensity (MFI).

Among participants with detectable poliovirus type 2–specific intestinal neutralization, the observed neutralizing antibody titers in stool were modest (Figure 1). Specifically, the geometric means of the peak poliovirus type 2–specific stool titers (ie, the highest titer observed per participant over the duration of follow-up) were 16.8 (range, 4–166) for the 6 participants with detectable intestinal neutralizing responses to nOPV2-c1 and 27.2 (7–84) for the 7 with detectable intestinal neutralizing responses to nOPV2-c2. Whereas the peak titers of poliovirus type 2–specific stool neutralization and IgA were closely correlated with each other (Spearman ρ = 0.69 for nOPV2-c1 [*P* = .005] and 0.62 for nOPV2-c2 [*P* = .01]), neither indicator was correlated significantly with the peak magnitudes of vaccine virus shedding (*P* > .05 for both) (Supplementary Figure 1).

Although not consistently statistically significant based on Spearman correlations, scatterplots suggest a potential positive trend between day 28 poliovirus type 2–specific serum neutralization and intestinal immune responses (Supplementary Figure 2). There was also evidence of a correlation of peak poliovirus type 2 stool IgA with peak IgA levels for poliovirus types 1 (Spearman ρ = 0.65 for nOPV2-c1 [*P* = .009] and 0.77 for nOPV2-c2 [*P* < .001]) and 3 (Spearman ρ = 0.38 for nOPV2-c1 [*P* = .16] and 0.76 for nOPV2-c2 [*P* = .001]).

## DISCUSSION

In this phase 1 clinical trial in a population of healthy IPV-immunized adults, we observed a modest but detectable rise in poliovirus type 2–specific intestinal mucosal neutralizing activity and IgA antibodies after administration of the 2 nOPV2 candidates. Despite a longer duration and higher magnitude of shedding in nOPV2-c1 versus nOPV2-c2 [7], the mucosal type 2–specific immune responses, like the serum neutralizing antibody responses [7], did not clearly differentiate the performance of the 2 vaccine candidates among the small number of participants evaluated in this study. nOPV2-c1 and nOPV2-c2 similarly induced detectable poliovirus type 2–specific intestinal neutralizing responses in 40.0% and 46.7% of participants, respectively. Peak titers of poliovirus type 2–specific stool neutralization and IgA correlated with each other but were not associated with the peak magnitudes of vaccine virus shedding.

These observations contribute to our growing understanding of vaccine-induced mucosal immunity to poliovirus across the life course. In young infants, primary vaccine series using only IPV induce negligible intestinal mucosal immunity to poliovirus [2, 3], and replication of Sabin OPV virus is not limited on subsequent challenge [12]. In contrast, vaccine schedules using OPV in infancy induce strong intestinal mucosal antibody responses and largely sterilizing immunity on OPV challenge [1]. Although there is evidence that OPV-induced intestinal immunity may wane significantly within a year of vaccination [13], a study of adults, aged 20–44 years, reported detecting mucosal IgA antibodies against all 3 serotypes of poliovirus in stool samples from 3 of the 11 participants who received OPV in childhood [14]. Furthermore, an investigation in adolescents, aged 16–18 years, found evidence of markedly lower frequencies of vaccine viral excretion in stool after OPV challenge in individuals who received OPV instead of IPV in childhood (excretion of OPV types 1, 2, and 3 after challenge, 20%, 24%, and 30%, respectively, for recipients with a history of OPV [with or without IPV] vs 50%, 69%, and 50% for those receiving only IPV [15]).

Building on this work, 2 recent investigations suggest the induction of mucosal immunity may be diminished if OPV is delivered beyond infancy. In a study of exclusively IPV-vaccinated children, aged 1–5 years, in Lithuania, who were challenged with 1 or 2 doses of mOPV2, only 32% of evaluable participants achieved a type 2–specific stool neutralizing titer ≥32 after the first dose of mOPV2 [6]. Despite having been previously vaccinated with 1 dose of mOPV2 and ≥3 doses of IPV, 32 of the 47 children (68%) receiving 2 doses of mOPV2 were reported to excrete virus after receipt of the second mOPV2 dose. Further evidence comes from an mOPV1 challenge study in adults, aged 18–50 years, in Sweden, who were vaccinated with 4 doses of IPV in childhood. Although each of the 12 evaluated participants had documented mOPV1 shedding after challenge, poliovirus type 1–specific neutralizing activity in stool samples remained low, never exceeding a titer of 18.4 for any of the participants during follow-up [4].

Although no direct comparisons of the responses can be made, and it remains possible that the nOPV2 candidates may induce lower levels of mucosal antibodies than the original Sabin OPV strains, the modest intestinal antibody responses observed in the current phase 1 trial in healthy adults are also consistent with our hypothesis of an age-related diminution in the mucosal immunogenicity of live attenuated poliovirus vaccines. Further research is needed to define the differences in mucosal immune responses to vaccination in children and adults. Potential mechanisms that warrant further investigation include oral tolerance, age-related immunosenescence, and an influence of IPV as initial immunogen. Furthermore, assay sensitivity in relation to age at stool collection remains an area of active investigation. The results from a subsequent phase II trial (NCT03554798)—which administered the nOPV2 candidates to healthy children, aged 1–5 years, previously vaccinated with bOPV-bOPV-bOPV + IPV in Panama—will be critical for understanding the role of age in the mucosal immunogenicity of the nOPV2 candidates.

In conclusion, these results contribute to our broader understanding of the induction of mucosal immunity. The modest but clear induction of mucosal immune responses to nOPV2s suggest the vaccines are likely to reduce cVDPV transmission under outbreak scenarios and strengthen the case for further clinical trials.

## Supplementary Data

Supplementary materials are available at The Journal of Infectious Diseases online. Consisting of data provided by the authors to benefit the reader, the posted materials are not copyedited and are the sole responsibility of the authors, so questions or comments should be addressed to the corresponding author.

jiaa783_suppl_Supplementary_Materials

## Notes

***Financial support.*** This work was supported by the Bill & Melinda Gates Foundation, the Wellcome Trust (204928/Z/16/Z to E. B. B.), and the Japan Agency for Medical Research and Development (20fk0108085j0002 to M. A.).

***Potential conflicts of interest.*** C. G. received personal consulting fees from BMGF during the primary study and A. S. B. is an employee of the BMGF, which provided support for the study. All other authors: No reported conflicts. All authors have submitted the ICMJE Form for Disclosure of Potential Conflicts of Interest. Conflicts that the editors consider relevant to the content of the manuscript have been disclosed.
