# Supplementary material for: Intestinal Antibody Responses to 2 Novel Live Attenuated Type 2 Oral Poliovirus Vaccines in Healthy Adults in Belgium
Source: J Infect Dis. 2020 Dec 24;226(2):287–91. doi: 10.1093/infdis/jiaa783 (PMC9400418; doi:10.1093/infdis/jiaa783)
Supplement: jiaa783_suppl_Supplementary_Materials [file jiaa783_suppl_supplementary_materials.docx]

**Supplementary Materials**

**A
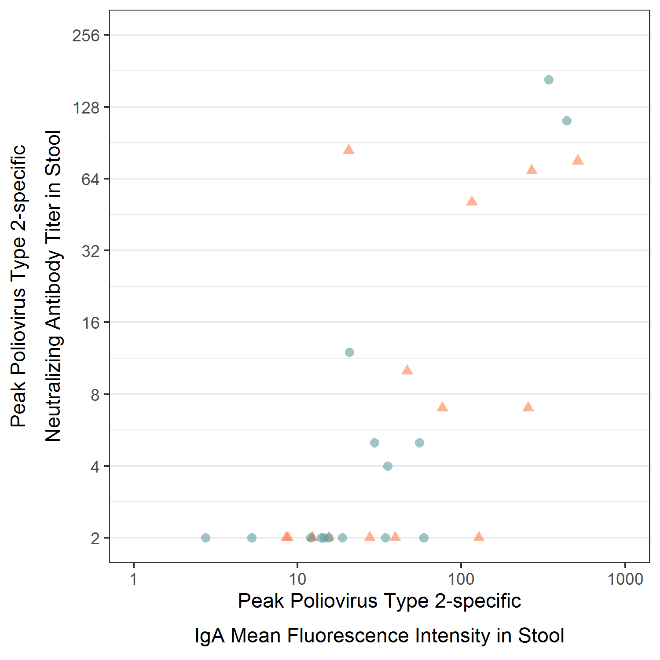
B
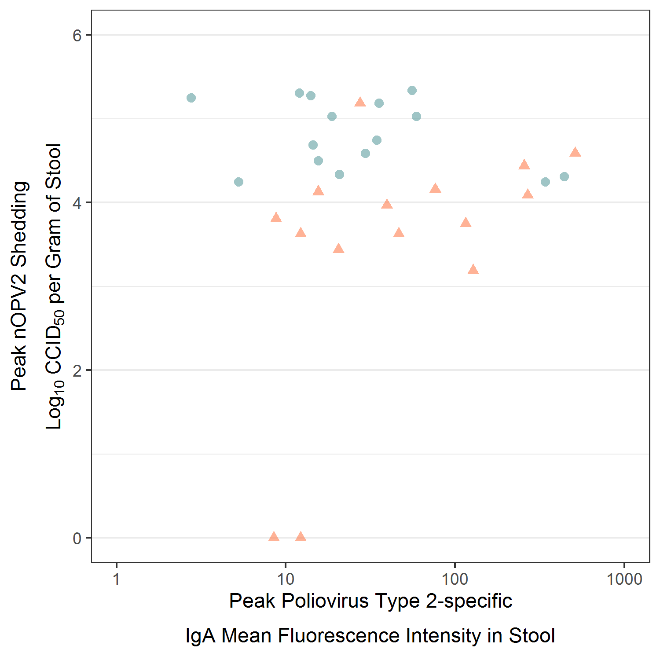
**

**C
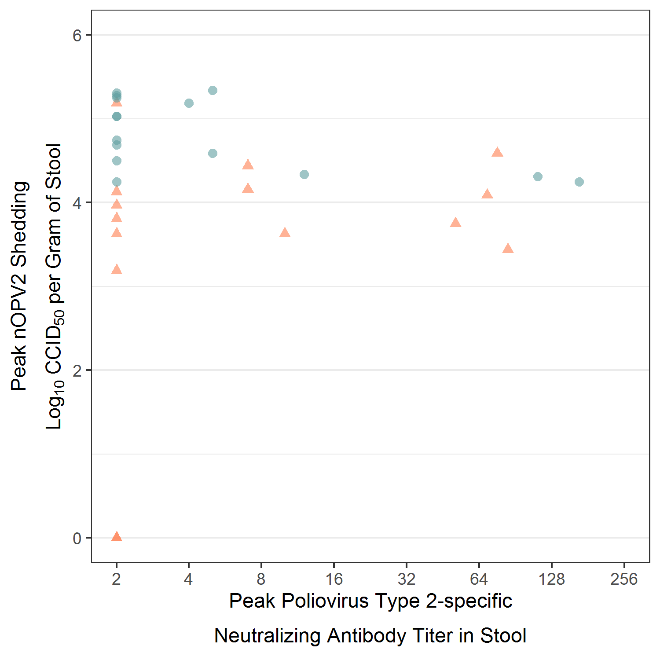
**

**Supplementary Figure 1.** Pairwise correlations between peak poliovirus type 2-specific stool IgA, neutralization, and nOPV2-c1 (blue circles) and nOPV2-c2 (orange triangles) shedding. (A) peak poliovirus type 2-specific stool IgA and peak poliovirus type 2-specific stool neutralization (Spearman’s rho: nOPV2-c1 = 0.69, p=0.005; nOPV2-c2 = 0.62, p=0.013), (B) peak poliovirus type 2-specific stool IgA and peak shedding after receipt of nOPV2 candidates (Spearman’s rho: nOPV2-c1 = -0.23, p=0.42; nOPV2-c2 = 0.50, p=0.058), and (C) peak poliovirus type 2-specific stool neutralization and peak shedding after receipt of nOPV2 candidates (Spearman’s rho: nOPV2-c1 = -0.38, p=0.16; nOPV2-c2 = 0.21, p=0.46).

**A
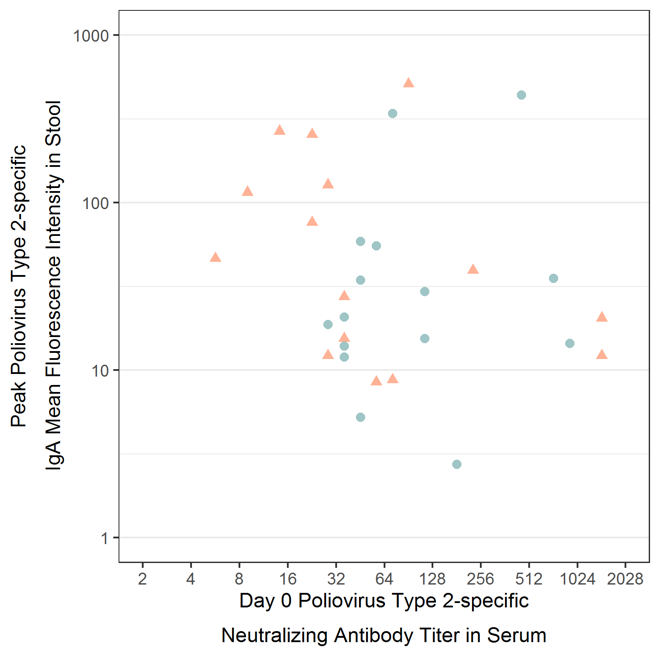
B
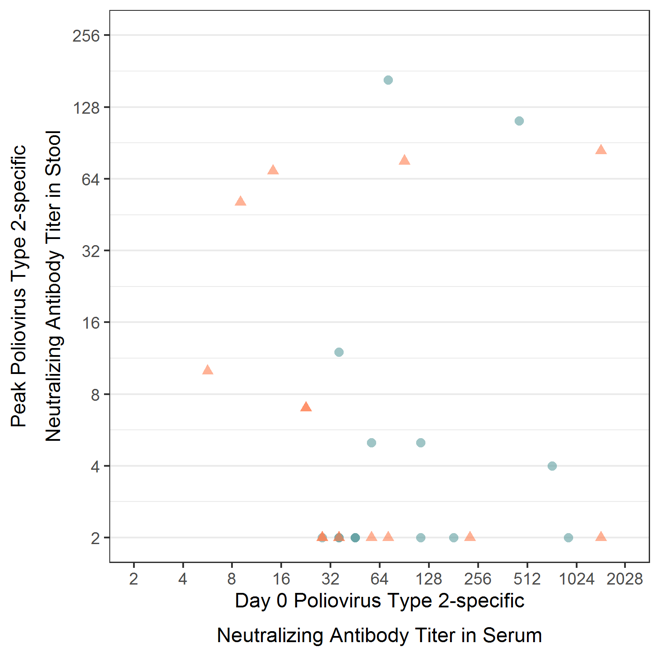
**

**C
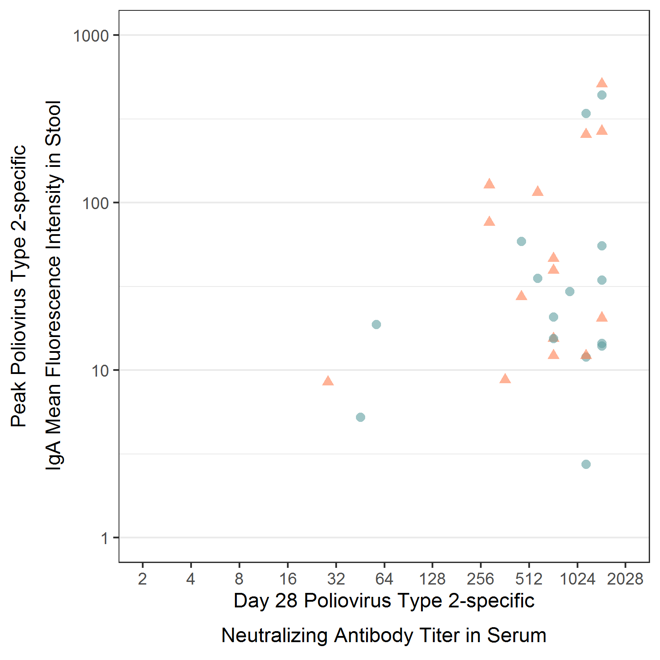
D
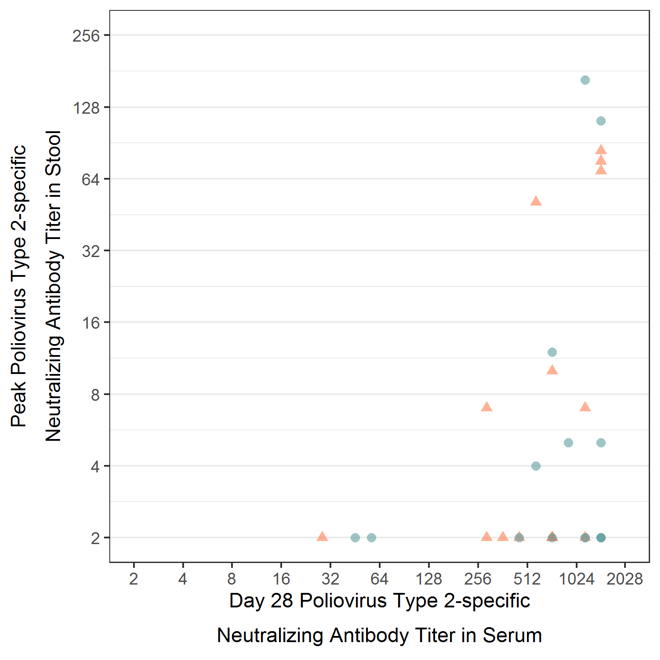
**

**Supplementary Figure 2.** Comparison of serum neutralization on days 0 and 28 with peak poliovirus type 2-specific stool IgA and peak poliovirus type 2-specific stool neutralization over follow-up. nOPV2-c1 (blue circles) and nOPV2-c2 (orange triangles). (A) day 0 poliovirus type 2-specific serum neutralization and peak poliovirus type 2-specific stool IgA (Spearman’s rho: nOPV2-c1=0.21, p=0.46; nOPV2-c2=-0.46, p=0.09), (B) day 0 poliovirus type 2-specific serum neutralization and peak poliovirus type 2-specific stool neutralization (Spearman’s rho: nOPV2-c1=0.25, p=0.37; nOPV2-c2=-0.24, p=0.39), (C) day 28 poliovirus type 2-specific serum neutralization and peak poliovirus type 2-specific stool IgA (Spearman’s rho: nOPV2-c1=0.14, p=0.61; nOPV2-c2=0.37, p=0.18), (D) day 28 poliovirus type 2-specific serum neutralization and peak poliovirus type 2-specific stool neutralization (Spearman’s rho: nOPV2-c1=0.17, p=0.55; nOPV2-c2=0.59, p=0.02).
